# A Hospital Villa for Bexley Mental Hospital

**Published:** 1912-07-20

**Authors:** 


### A Hospital Villa for Bexley Mental Hospital.

Plans have been prepared, subject to the approval of the Home Secretary, for a hospital villa to accommodate thirty male patients suffering from dysentery and phthisis at Bexley Mental Hospital. At present considerable difficulty is experienced at Bexley in the treatment of phthisical and dysenteric male patients largely because of insufficient facilities for supervision. Such male patients are now housed in the East Villa, which was built specially for chronic cases and is consequently quite unsuitable for its present occupants. Moreover, the provision for open-air treatment is felt to be inadequate. The proposed villa is designed to give patients open-air treatment, while conforming in its chief features to the recognised villa type. A folding partition will permit the south front or any section of it to be thrown open, and a cross partition will allow the building to be divided into two or three sections, or of its being used as a common hospital. Eighteen phthisical and twelve dysenteric patients will be accommodated. The total cost of providing and equipping the hospital is expected to be £5,000.

